# Alteration in yield and oil quality traits of winter rapeseed by lodging at different planting density and nitrogen rates

**DOI:** 10.1038/s41598-017-18734-8

**Published:** 2018-01-12

**Authors:** Shahbaz Khan, Sumera Anwar, Jie Kuai, Ali Noman, Muhammad Shahid, Mairaj Din, Ahmed Ali, Guangsheng Zhou

**Affiliations:** 1 College of Plant Science and Technology, Huazhong Agricultural University, Wuhan, Hubei Province P.R. China; 2 Government College University, Faisalabad, Pakistan; 3 College of Resources and Environment, Huazhong Agricultural University, Wuhan, Hubei Province P.R. China

## Abstract

Lodging is a factor that negatively affects yield, seed quality, and harvest ability in winter rapeseed (*Brassica napus* L.). In this study, we quantified the lodging-induced yield losses, changes in fatty acid composition, and oil quality in rapeseed under different nitrogen application rates and planting densities. Field experiments were conducted in 2014–2017 for studying the effect of manually-induced lodging angles (0°, 30°, 60°, and 90°), 10, 20 and 30 d post-flowering at different densities and nitrogen application rates. The fertilization/planting density combination N_270_D_45_ produced the maximum observed yield and seed quality. Timing and angle of lodging had significant effects on yield. Lodging at 90° induced at 10 d post-flowering caused the maximum reduction in yield, biomass, and silique photosynthesis. Seed yield losses were higher at high N application rates, the maximum being at N_360_D_45_. Lodging decreased seed oil content and altered its fatty acid composition by increasing stearic and palmitic acid content, while decreasing linoleic and linolenic acid content, and deteriorating oil quality by increasing erucic acid and glucosinolate content. Therefore, lodging-induced yield loss and reduction in oil content might be reduced by selecting optimum N level and planting density.

## Introduction

Rapeseed (*Brassica napus* L.) is the most extensively cultivated oil crop and the fourth main crop in China after rice, wheat, and maize^1^. China is the second largest rapeseed producer after Canada, accounting for almost one-fourth of the world’s total production and planting area^2^; with total area of cultivation reaching 7.59 million hectares in 2015^3^. Yangtze River Basin is largest rapeseed producing belt in China where an intensive crop production system is commonly used by farmers to obtain high yields^4^. In the last decade, the yield and quality of rapeseed have rapidly increased owing to mechanized harvesting, the introduction of high-yield cultivars, and the adoption of improved agronomic practices. However, a further increase in rapeseed yield is necessary to meet the rising demand for edible oil given population growth and increasing economic development^1^.

Conventional rapeseed harvesting is becoming less common because it is costly and labor intensive compared with mechanical harvesting. Owing to labor scarcity in rural areas and better mechanization, the rapeseed industry has started to transition from manual to mechanized farming. In the past five years, the use of new and precise machines for seeding and harvesting, as well for plant density adjustment, weed management, and controlled-release fertilizers, have significantly increased the planting area, total production, and yield per unit area of rapeseed^1^.

Lodging negatively affects the quality of rapeseed and constrains mechanical harvesting. In lodged plants, the shoot is displaced from an upright position, which increases the risk of losses from diseases in humid areas and hampers harvesting operations^5^. Lodging mostly occurs before or at the beginning of grain filling, decreasing seed yield and quality. Several studies reported that lodging at the early reproductive stages such as during flowering or early pod development resulted in greater yield losses^6,7^. However, other studies showed that lodging at the later growth stages caused higher reductions in yield. For instance, in soybean, artificial lodging at the flowering stage decreased yield by 9% due to the development of new branches, whereas at the pod maturity stage, lodging decreased yield by 34%^8^. In wheat, natural and artificially induced lodging caused yield losses of 0–80%, which increased as the lodging degree increased from 45° to 80°^9^. In rice, photosynthesis was reduced by 60–80% from lodging^10^.

Lodging usually occurs as a result of interactions between the plant (stem diameter, cell wall composition, and root depth) and the environment (wind, rain, and soil structure) as well as agronomic factors (plant density, use of nitrogen [N] fertilizers, and water management)^11^. Plant density affects yield and the extent of lodging by altering the crop canopy^12^. High plant densities enhance light capture by the canopy and increase yield up to a certain saturation threshold^13^. At high plant densities, the canopy is uniform, stems are thinner, branches are shorter, and maturation is more synchronized, all of which are factors that facilitate mechanized harvesting and decrease yield losses^14,15^.

Rapeseed production strongly depends on the application of chemical fertilizers, especially N, which plays an important role in increasing yield^16,17^. However, the response of rapeseed to N fertilization depends on many environmental factors. The excessive use of N increases the risk of lodging by increasing the height and centre of gravity of the plants and by decreasing stem diameter and the cell wall thickness of basal internodes^18,19^. Therefore, it is crucial to identify the optimum dose of N fertilizer to improve the management of winter rapeseed.

Grain and oil quality traits, such as oil content, protein content, and fatty acid composition, have received much attention from rapeseed breeders. Fatty acid composition and the level of unsaturated fatty acids are controlled genetically but are also affected by the environment^20^. Previous reports indicated that salinity^21^, irrigation, drought^22^, temperature^20^, and shading stress^23^ induced changes in fatty acid composition. For instance, oilseed plants grown under shaded conditions produced seeds with a lower level of fatty acids than those grown under full sun^23^. However, information on lodging and the induced changes in fatty acid composition and oil quality traits are limited in rapeseed. Therefore, this study aimed to elucidate the relationships of yield and oil composition with different angles of lodging, plant densities, and N rates.

## Results

### Humidity and air temperature

**Figure 1:**
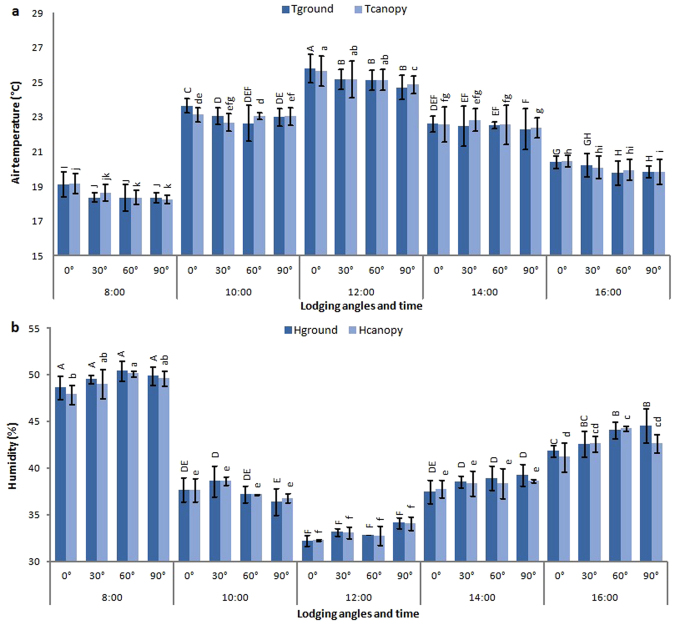
Air temperature (**a**) and humidity (**b**) recorded at different times of day and at lodging angles (0, 30, 60 and 90) during pod filling stage in rapeseed field. Tground: air temperature at ground level; Tcanopy: canopy air temperature; Hground: humidity at ground level; Hcanopy: humidity at canopy level. Different letters indicate significant differences at p < 0.05.

Maximum air temperature and minimum humidity at the ground and canopy level of plants was recorded at noon (Fig. 1). Air temperature at canopy and ground level of rapeseed was decreased with increasing the angle of lodging. In contrast to air temperature, relative humidity was increased in lodged plants as compared to unlodged plants.

### Changes in yield and yield components in relation to lodging stage, plant density, and N rate

The lodging stage showed a significant effect on seed yield, seed weight per plant, and silique weight, but not on the number of siliques per plant and the 1,000 seed weight (Table 1). Lodging induced at S_10_ caused a lower seed weight per plant, 1000 seed weight, silique weight, and seed yield compared with that at S_20_ and S_30_. Lodging induced at S_10_ caused more yield loss (5.8–12.6%) than at S_20_ (2.9–8.4%) and S_30_ (0–2.1%).Seed yield increased as the plant density and N rate increased, reaching a maximum at N_270_D_45_, but it showed no further increase with additional N. At N_360,_ increasing the density from 30 to 45 plants m^−2^ decreased the seed yield. The silique weight and number of siliques per plants increased with increasing N rate but decreased with increasing density. Maximum silique weight and silique numbers were observed at N_360_D_15_.

**Table 1:** Effect of lodging stages (10 d post flowering, S_10_; 20 d post flowering, S_20_; and 30 d post flowering, S_30_), plant densities (D_15_, 15 plants m^−2^; D_30_, 30 plants m^−2^; and D_45_, 45 plants m^−2^), and nitrogen rates (N_180_, 180 kg N ha^−1^; N_270_, 270 kg N ha^−1^; and N_360_, 360 kg N ha^−1^) on seed yield, seed weight, silique weight, and silique number of rapeseed during the growing seasons of 2014/2015.

Treatments			Seed yield (t ha^−1^)	Yield loss (%)	Seeds weight (g plant^−1^)	1000 seed weight (g)	Silique weight (g plant^−1^)	Silique no. (plant^−1^)
Stage	Nitrogen	Density						
S_10_	N_180_	D_15_	1.54	9.94	10.80	2.78	9.48	236.7
		D_30_	1.84	7.54	9.67	2.67	6.82	211.3
		D_45_	2.07	11.73	8.29	2.44	6.03	66.3
	N_270_	D_15_	1.72	11.21	11.02	3.17	9.48	244.7
		D_30_	2.26	5.83	8.68	2.97	7.01	220.7
		D_45_	2.56	7.14	7.05	2.76	6.51	91.3
	N_360_	D_15_	2.40	6.25	12.31	3.33	9.67	254.0
		D_30_	2.42	6.92	9.63	2.80	9.10	233.0
		D_45_	2.08	12.61	7.67	2.56	9.22	198.0
S_20_	N_180_	D_15_	1.57	8.44	10.82	3.10	9.74	240.7
		D_30_	1.93	3.08	9.67	2.87	8.07	117.0
		D_45_	2.22	5.46	8.29	2.71	6.58	62.3
	N_270_	D_15_	1.85	4.64	11.02	3.22	11.76	254.3
		D_30_	2.33	2.92	8.68	2.93	9.07	239.3
		D_45_	2.68	2.90	7.05	2.80	6.96	130.7
	N_360_	D_15_	2.39	6.71	11.30	3.39	10.32	260.3
		D_30_	2.51	3.46	10.63	2.97	9.90	248.0
		D_45_	2.24	5.84	8.67	2.61	8.28	226.3
S_30_	N_180_	D_15_	1.68	1.75	12.15	3.50	10.50	239.0
		D_30_	1.98	0.50	9.86	2.83	9.93	163.0
		D_45_	2.34	0.43	7.45	2.52	7.98	64.1
	N_270_	D_15_	1.90	2.06	11.73	3.89	12.87	250.0
		D_30_	2.40	0.00	10.82	3.00	13.14	231.0
		D_45_	2.74	0.72	8.22	2.83	10.57	113.0
	N_360_	D_15_	2.46	3.91	11.08	3.86	13.51	256.0
		D_30_	2.60	0.00	9.77	3.22	11.86	238.3
		D_45_	2.38	0.10	15.15	2.34	11.60	211.5
LSD_0.05_			0.33	—	1.86	0.17	3.76	45.9
Stage (S)			***	—	**	ns	***	ns
Nitrogen (N)			***	—	***	*	*	*
Density (D)			**	—	***	***	**	***
S × N × D			ns	—	ns	*	ns	*

ns, not significant; *, ** and ***, significant at *P* < 0.05, 0.01 and 0.001, respectively, n = 4.

### Plant height, aboveground dry biomass, root dry biomass, and root neck diameter in relation to lodging stage, plant density, and N rate

Lodging induced at S_10_ caused a significant reduction in aboveground dry biomass compared with lodging induced at S_20_ and S_30_ (Table 2). Plant height, root neck diameter, and root dry biomass showed no significant differences under different lodging stages. Plant height significantly increased with an increase in N and decreased by increasing density. Minimum plant height was recorded at N_180_D_45_ and maximum height was observed at N_360_D_15_. Aboveground dry biomass increased as N increased, whereas root neck diameter and root dry biomass were lower at high N rates compared with those at low N rates. Root neck diameter, and root dry biomass significantly decreased linearly with increasing plant density.

**Table 2:** Effect of lodging stages (10 d post flowering, S_10_; 20 d post flowering, S_20_; and 30 d post flowering, S_30_), plant densities (D_15_, 15 plants m^−2^; D_30_, 30 plants m^−2^; and D_45_, 45 plants m^−2^), and nitrogen rates (N_180_, 180 kg N ha^−1^; N_270_, 270 kg N ha^−1^; and N_360_, 360 kg N ha^−1^) on plant growth in rapeseed during the growing seasons of 2014/2015.

Treatments			Plant height (cm)	Root neck diameter (mm)	Aboveground dry biomass (g)	Root dry biomass (g)
Stage	Nitrogen	Density				
S_10_	N_180_	D_15_	157.4	13.45	10.47	14.21
		D_30_	148.9	16.46	6.29	7.9
		D_45_	135.0	13.66	5.83	6.58
	N_270_	D_15_	162.5	16.81	15.83	14.56
		D_30_	158.3	14.79	10.77	9.91
		D_45_	139.5	13.13	6.67	5.66
	N_360_	D_15_	172.7	11.41	18.11	7.54
		D_30_	171.3	12.19	15.34	7.51
		D_45_	151.7	13.28	8.44	6.26
S_20_	N_180_	D_15_	152.1	14.49	5.70	16.89
		D_30_	148.8	14.95	13.34	4.23
		D_45_	137.6	13.48	9.24	8.38
	N_270_	D_15_	165.4	15.27	19.51	12.71
		D_30_	157.0	13.25	13.36	5.1
		D_45_	144.1	11.83	11.06	6.01
	N_360_	D_15_	180.5	12.83	20.40	7.71
		D_30_	164.9	14.77	15.74	4.21
		D_45_	163.1	12.99	20.59	4.97
S_30_	N_180_	D_15_	150.5	15.09	10.09	10.55
		D_30_	142.2	13.00	11.34	5.76
		D_45_	143.5	12.95	8.24	5.48
	N_270_	D_15_	162.6	13.43	17.88	12.84
		D_30_	157.6	13.74	14.36	11.93
		D_45_	153.5	10.72	10.15	10.11
	N_360_	D_15_	171.3	13.39	17.26	8.77
		D_30_	168.5	12.25	19.24	7.36
		D_45_	160.9	10.30	15.95	4.3
LSD_0.05_			13.19	3.57	7.50	4.54
Stage (S)			ns	ns	*	ns
Nitrogen (N)			***	*	***	**
Density (D)			***	*	ns	***
S × N × D			ns	ns	ns	ns

ns, not significant; *, ** and ***, significant at *P* < 0.05, 0.01 and 0.001, respectively, n = 4.

### Changes in yield and yield components in relation to lodging angle, plant density, and N rate

Lodging significantly decreased seed yield and seed weight per plant compared with the control (Table 3). The reduction in yield and yield components was greater with increasing lodging angle, reaching a maximum at 90°. The plants that lodged at 90° showed a seed yield loss of 14–28% in 2015/16 and 19–32% in 2016/17 compared with the control. In lodged plants, more yield loss was observed at higher densities and N rates, with a maximum yield loss recorded at N_360_D_45_.

**Table 3:** Effect of different induced lodging angles (0°, 30°, 60°, and 90°), plant densities (D_15_, 15 plants m^−2^; D_30_, 30 plants m^−2^; and D_45_, 45 plants m^−2^), and nitrogen rates (N_180_, 180 kg N ha^−1^; N_270_, 270 kg N ha^−1^; and N_360_, 360 kg N ha^−1^) on seed weight, silique weight, silique number, and seed yield of rapeseed during the growing seasons of 2015/2016 and 2016/2017.

Treatments			Seed yield (t ha^−1^)				Seed yield loss (%)			Seeds weight (g plant^−1^)			
Year	Nitrogen	Density	0°	30°	60°	90°	30°	60°	90°	0°	30°	60°	90°
2015/16	N_180_	D_15_	1.71	1.61	1.40	1.38	5.8	18.1	19.3	13.28	11.82	9.93	8.79
		D_30_	1.99	1.77	1.62	1.54	11.1	18.6	22.6	11.67	10.73	8.67	7.58
		D_45_	2.35	2.12	1.83	1.75	9.8	22.1	25.5	10.57	9.29	7.45	7.39
	N_270_	D_15_	1.94	1.90	1.81	1.66	2.1	6.7	14.4	15.96	15.02	13.29	12.17
		D_30_	2.40	2.29	1.91	1.85	4.6	20.4	22.9	13.53	12.68	10.03	9.67
		D_45_	2.76	2.54	2.22	2.09	8.0	19.6	24.3	11.82	11.25	9.81	8.09
	N_360_	D_15_	2.56	2.36	1.94	1.89	7.8	24.2	26.2	16.39	15.32	13.22	11.12
		D_30_	2.60	2.45	2.09	1.91	5.8	19.6	26.5	14.29	13.63	11.16	10.4
		D_45_	2.28	2.06	1.71	1.64	9.6	25.0	28.1	11.48	10.67	10.35	8.85
2016/17	N_180_	D_15_	1.55	1.41	1.28	1.25	9.0	17.4	19.4	13.51	11.15	10.96	9.80
		D_30_	1.95	1.62	1.59	1.55	16.9	18.5	20.5	12.19	10.63	9.29	8.84
		D_45_	2.32	1.92	1.86	1.79	17.2	19.8	22.8	10.47	9.42	7.57	7.14
	N_270_	D_15_	1.66	1.43	1.40	1.27	13.9	15.7	23.5	15.71	14.76	13.02	12.03
		D_30_	2.29	1.94	1.85	1.72	15.3	19.2	24.9	14.44	12.34	11.01	10.66
		D_45_	3.02	2.65	2.61	2.32	12.3	13.6	23.2	13.07	11.27	10.39	9.34
	N_360_	D_15_	2.56	2.15	1.89	1.85	16.0	26.2	27.7	15.87	15.28	13.63	11.55
		D_30_	2.72	2.46	2.20	2.02	9.6	19.1	25.7	15.39	12.74	9.18	9.46
		D_45_	2.24	1.84	1.63	1.53	17.8	27.2	31.7	13.95	10.28	8.39	8.18
Year (Y)			ns					—		ns			
Lodging (L)			***					—		***			
Nitrogen (N)			***					—		**			
Density (D)			***					—		***			
Y × L × N × D			*					—		ns			

ns, not significant; *, ** and ***, significant at *P* < 0.05, 0.01 and 0.001, respectively, n = 4.

Lodging significantly decreased silique weight and number of siliques (Table 4). At N_360_D_15_, plants that lodged at 90° showed a reduction of 46 and 47% in silique weight, and 28 and 30% in the number of siliques per plant, during 2015/16 and 2016/17, respectively.

**Table 4:** Effect of different induced lodging angles (0°, 30°, 60°, and 90°), plant densities (D_15_, 15 plants m^−2^; D_30_, 30 plants m^−2^; and D_45_, 45 plants m^−2^), and nitrogen rates (N_180_, 180 kg N ha^−1^; N_270_, 270 kg N ha^−1^; and N_360_, 360 kg N ha^−1^) on silique weight, silique numbers and aboveground dry weight of rapeseed during the growing seasons of 2015/2016 and 2016/2017.

Treatments			Silique weight (g plant^−1^)				Silique no. (plant^−1^)				Plant height (cm)			
Year	Nitrogen	Density	0°	30°	60°	90°	0°	30°	60°	90°	0°	30°	60°	90°
2015/16	N_180_	D_15_	14.99	11.22	9.48	8.97	249.8	237	220.4	176.4	157.4	150.7	152.4	151.6
		D_30_	12.13	10.31	7.82	7.62	233.6	211.3	199.6	167.1	144.7	148.9	151.9	149.3
		D_45_	11.74	8.64	7.03	6.73	221.3	103.6	203.7	155.2	134.2	135.0	141.5	140.7
	N_270_	D_15_	14.63	13.89	9.48	7.27	265.8	244.7	229.6	188.2	164.8	162.5	156.0	155.0
		D_30_	13.95	11.35	7.01	7.54	252.8	220.7	190.5	170.4	166.8	158.3	166.3	150.1
		D_45_	12.93	9.37	6.51	6.55	241.7	116.7	183.7	177.2	160.5	139.5	162.2	149.7
	N_360_	D_15_	16.78	13.50	9.67	8.77	274.2	260	233.7	196.3	181.6	171.3	171.7	167.0
		D_30_	15.35	14.62	9.10	7.68	262.7	233	212.5	194.8	167.8	172.7	161.7	150.2
		D_45_	12.71	11.03	9.22	6.10	250.2	198	178.8	173.6	162.9	151.7	141.9	139.8
2016/17	N_180_	D_15_	14.92	12.71	9.75	9.88	241.6	240.7	195.7	166.2	164.7	155.9	157.9	155.6
		D_30_	14.31	11.12	9.22	9.03	228.5	117	199.4	140.6	145.7	145.2	147.8	142.7
		D_45_	12.57	9.89	8.83	7.85	230.1	62.3	166.8	133.9	136.4	136.2	143.6	138.4
	N_270_	D_15_	18.71	15.75	10.88	10.8	257.3	254.3	220.5	170.7	167.2	160.1	164.0	151.9
		D_30_	17.88	12.73	10.92	7.79	244.4	239.3	188.1	166.3	153.1	141.6	152.2	149
		D_45_	15.98	10.52	10.75	9.47	230.3	130.7	171.6	152.3	148.8	140.3	135.0	148.4
	N_360_	D_15_	20.04	17.32	15.84	10.81	268.5	257.2	228.1	188.2	177.0	173.5	170.3	170.7
		D_30_	19.04	14.57	14.47	10.2	258.2	248	202.4	156.6	168.1	160.4	155.8	158.8
		D_45_	14.13	13.45	12.37	9.77	248.7	226.3	180	163.3	152.6	168.7	144.8	149.9
Year (Y)			**				ns				ns			
Lodging (L)			***				***				ns			
Nitrogen (N)			*				**				***			
Density (D)			***				**				ns			
Y × L × N × D			ns				ns				ns			

ns, not significant; *, ** and ***, significant at *P* < 0.05, 0.01 and 0.001, respectively, n = 4.

### Plant height, aboveground dry biomass, root dry biomass, and root neck diameter in relation to lodging angle, plant density, and N rate

Plant height significantly increased with an increase in N; however, the effects of lodging angles and density rates were not significant for plant height (Table 4). Aboveground dry biomass increased as N increased, whereas root neck diameter and root dry biomass were lower at high N rates compared with those at low N rates (Table 5). Root neck diameter, and root dry biomass significantly decreased linearly with increasing plant density. Lodging significantly reduced aboveground dry biomass whereas other growth variables, root neck diameter, and root dry biomass remained unaffected by lodging angles.

**Table 5:** Effect of different induced lodging angles (0°, 30°, 60°, and 90°), plant densities (D_15_, 15 plants m^−2^; D_30_, 30 plants m^−2^; and D_45_, 45 plants m^−2^), and nitrogen rates (N_180_, 180 kg N ha^−1^; N_270_, 270 kg N ha^−1^; and N_360_, 360 kg N ha^−1^) on plant height, root neck diameter and root dry biomass of rapeseed during the growing seasons of 2015/2016 and 2016/2017.

Treatments			Aboveground dry biomass (g)				Root neck diameter (mm)				Root dry biomass (g)			
Year	Nitrogen	Density	0°	30°	60°	90°	0°	30°	60°	90°	0°	30°	60°	90°
2015/16	N_180_	D_15_	12.72	11.47	11.02	11.61	16.29	16.46	14.72	15.69	14.45	14.56	12.77	12.49
		D_30_	10.77	9.73	8.26	8.97	16.17	13.66	12.17	14.27	12.50	13.51	11.72	11.09
		D_45_	9.01	8.833	8.11	7.29	14.05	13.45	11.41	11.35	11.11	9.54	9.61	8.98
	N_270_	D_15_	18.11	17.05	12.41	11.65	15.99	16.81	16.88	13.98	13.88	14.21	12.44	12.56
		D_30_	15.29	15.31	10.34	10.47	15.63	14.79	14.00	11.04	9.58	9.91	11.01	9.17
		D_45_	15.16	12.67	6.09	6.19	13.21	13.13	12.43	9.63	8.80	7.66	8.23	8.11
	N_360_	D_15_	18.64	18.34	15.36	14.04	15.94	13.28	15.82	12.43	12.36	10.90	11.01	11.42
		D_30_	15.83	15.25	14.86	15.03	15.32	12.19	12.91	13.43	8.55	8.58	7.40	7.82
		D_45_	11.2	8.44	9.5	9.55	10.90	11.41	10.74	9.71	8.69	6.26	7.48	8.01
2016/17	N_180_	D_15_	13.76	12.84	12.62	10.28	15.84	15.94	15.31	14.82	13.38	11.21	11.63	12.33
		D_30_	12.13	11.92	10.54	10.5	13.93	13.15	13.19	13.35	11.74	10.71	9.16	10.66
		D_45_	7.61	8.34	8.02	7.50	13.01	10.92	13.99	11.03	9.17	8.57	9.09	9.24
	N_270_	D_15_	14.14	15.81	11.33	10.51	14.34	13.57	12.85	13.76	13.26	11.37	12.97	11.13
		D_30_	15.83	12.3	12.05	10.74	13.80	11.73	12.55	12.32	9.28	8.14	9.30	9.99
		D_45_	14.12	9.82	9.05	6.2	12.86	12.50	10.57	11.83	7.84	7.80	8.07	7.36
	N_360_	D_15_	16.54	15.87	12.33	11.59	12.32	12.47	11.37	13.76	11.61	9.63	9.61	10.36
		D_30_	15.34	15.1	12.15	10.22	11.99	12.03	11.16	11.97	7.42	9.00	8.12	9.81
		D_45_	12.78	13.11	8.32	9.77	10.09	9.88	10.94	11.85	6.40	7.19	7.36	8.69
Year (Y)			ns				*				ns			
Lodging (L)			*				ns				ns			
Nitrogen (N)			***				ns				*			
Density (D)			ns				**				**			
Y × L × N × D			ns				ns				*			

ns, not significant; *, ** and ***, significant at *P* < 0.05, 0.01 and 0.001, respectively, n = 4.

### Silique photosynthesis in relation to lodging angle, plant density, and N rate

**Figure 2:**
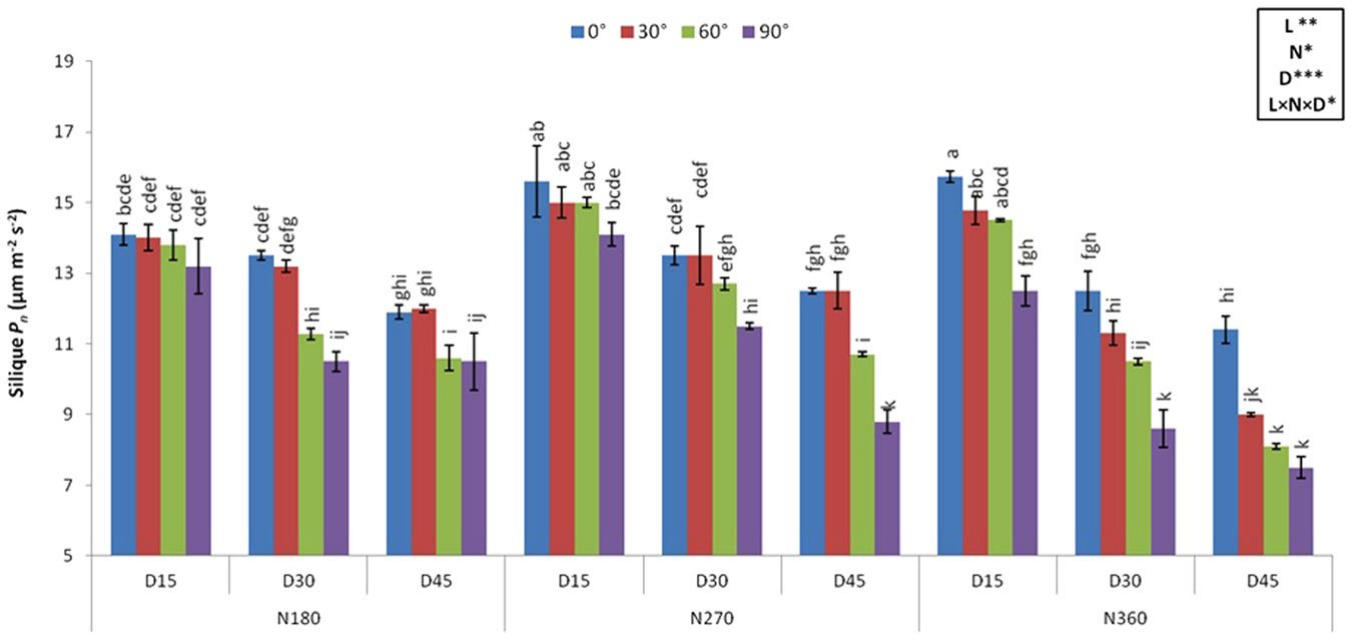
Silique photosynthesis (*P*_*n*_) at different induced lodging angles (0°, 30°, 60° and 90°), planting density and nitrogen rates. *, ** and *** indicate significance at *P* < 0.05, 0.01 and 0.001, respectively. Different letters indicate significant differences at p < 0.05. D15, D30 and D45 indicate 15, 30 and 45 plants m^−2^; N180, N270 and N360 are 180, 270 and 360 kg N ha^−1^ respectively.

Silique photosynthesis (*P*_*n*_) was significantly affected by lodging angles; decreased linearly with the increasing lodging angle (Fig. 2). Silique photosynthesis was significantly reduced with increasing plant density from D_15_ to D_45_ within the same N rate. At low density rates (D_15_), photosynthesis was increased with increasing N rate reaching a maximum at N_360,_ however at higher density, increasing N rate decreased silique photosynthesis. Overall maximum silique *P*_*n*_ was recorded in N_360_D_15_ at 0° (supported control) and minimum at N_360_D_45_ at 90°.

### Seed quality traits and fatty acid composition in relation to lodging stage, lodging angle, plant density, and N rate

Oil content significantly decreased with increasing lodging angle (Table 6), reaching a maximum reduction of 4.8% at 90° compared with the control. Oil content was not affected by plant density and decreased as N increased. The protein, glucosinolate, erucic acid, and saturated fatty acid (stearic, palmitic, and arachidic) contents tended to increase as the lodging angle increased, whereas the opposite trend was observed for the unsaturated fatty acid (linoleic and linolenic) content. Contents of erucic and arachidic acids were significantly reduced at high plant densities compared with those at low plant densities. However, the contents of stearic, palmitic, linoleic, and linolenic acids were not significantly affected by plant density. The protein and stearic acid contents increased significantly as N increased, whereas the opposite trend was observed for the glucosinolate and linoleic acid contents.

**Table 6:** Response of oil content, protein, glucosinolate, erucic acid and fatty acid composition in rapeseed at different induced lodging angles (0°, 30°, 60°, and 90°), plant densities (D_15_, 15 plants m^−2^; D_30_, 30 plants m^−2^; and D_45_, 45 plants m^−2^), and nitrogen rates (N_180_, 180 kg N ha^−1^; N_270_, 270 kg N ha^−1^; and N_360_, 360 kg N ha^−1^).

Treatments	Oil content (%)	Crude protein (%)	Glucosinolate (µmole g^−1^)	Erucic acid (%)	Oleic (18:1) (%)	Linoleic (18:2) (%)	Linolenic (18:3) (%)	Stearic (18:0) (%)	Palmitic (16:0) (%)	Arachidic (20:0) (%)
Lodging angle	*	**	*	*	ns	ns	*	*	ns	**
0°	41.4 a	25. 24 cd	31.3 b	1.13 bc	58.8 a	20.8 a	8.03 ab	1.48 b	4.41 b	3.67 b
30°	41.1 a	25.54 bc	31.3 b	1.19 abc	58.7 ab	20.78 ab	7.95 b	1.49 ab	4.46 ab	3.60 b
60°	39.6 b	25.84 ab	32.4 ab	1.32 ab	58.7 ab	20.69 ab	7.97 ab	1.52 a	4.49 a	3.75 ab
90°	39.4 b	25.99 a	33.2 a	1.49 a	58.3 b	20.51 ab	7.75 c	1.53 a	4.51 a	3.90 a
Plant density	ns	***	ns	***	***	ns	ns	ns	ns	*
D_15_	40.4 a	26.51 a	32.5 a	1.46 a	57.3 b	20.78 a	7.95 a	1.50 a	4.49 a	3.8 a
D_30_	39.7 a	25.05 b	32.1 a	1.05 b	58.9 a	20.56 a	8.01 a	1.49 a	4.46 a	3.68 a
D_45_	39.0 a	24.25 c	31.7 a	1.05 b	59.1 a	20.57 a	7.94 a	1.49 a	4.45 a	3.72 a
Nitrogen rate	***	***	**	ns	ns	***	**	***	ns	ns
N_180_	40.6 a	24.16 c	33.2 ab	1.27 a	58.3 a	20.98 a	7.9 b	1.45 b	4.45 a	3.67 b
N_270_	40.5 a	24.83 b	32.1 b	1.15 a	58.4 a	20.81 a	8.09 a	1.51 a	4.47 a	3.89 b
N_360_	38.6 b	26.83 a	31.0 c	1.49 a	58.7 ab	20.18 b	7.9 b	1.52 a	4.49 a	3.62 b

Values in a column followed by the same letter are not significantly different at P < 0.05 as determined by the LSD test; n = 27. ns, non-significant; *, ** and ***, significant at *P* < 0.05, 0.01 and 0.001, respectively.

## Discussion

In this study, we investigated the effects of lodging induced at the late growth stages of rapeseed on yield, biomass, silique photosynthesis, seed oil content, and fatty acid composition of rapeseed. Results from present study indicate that the reduction in yield by lodging varies with the timing and angle of lodging. The seed yield losses increased with increase in lodging angle, reaching a maximum reduction of 19–26% at 90° during early pod filling as compared with that in the control (Table 3). Similarly Kendall *et al*.^24^ reported that the crop developmental stage at which lodging occurred, and the angle of lodging had significant effects on yield. Moreover they reported that lodging to 90° during flowering reduced the yield by 46% and lodging to 45° during flowering reduced the yield by approximately 20%^24^. Lodging-induced yield losses are related to reductions in the number of seeds per unit area. Accordingly, our results depicts that the lodging significantly reduced the seed weight per plant, number of silique/plant and weight of silique/plant, which ultimately reduced seed yield (Tables 3 and 4). Reduction in light interception and photosynthesis in lodged crop and pre-harvest yield loss by pod shattering might be possible reasons underlying seed yield losses.

Our study in 2014/15 indicated that the 1000 seed weight was not affected by lodging stages, whereas seed weight per plant and silique weight were significantly affected by lodging stage. In addition, more reduction occurred when lodging was imposed 10 d post-flowering as compared with the later stages. Therefore, it indicated that lodging at the early pod filling stage caused yield losses by reducing the number of seeds rather than affecting the individual seed weight. A similar study on soybean indicated that the lodging induced at the full pod stage affected the number of seeds per plant more than seed size^25^. Therefore, decrease in seed yield was primarily due to reduction in number of seeds produced on lodged plants

Baylis and Wright^5^ reported that lodging induced at early pod filling stages caused higher yield losses as compared with that caused by lodging induced at the end of the flowering stage. Moreover, they reported that pre-harvest seed loss and yield were negatively correlated. In contrast, Kendall *et al*.^24^ reported that lodging induced at the flowering stage caused more yield loss in rapeseed and a small amount of yield was lost when lodging occurred during grain filling.

Information about physiological mechanism of lodging-related yield losses in rapeseed is limited. Berry and Spink^26^ reported that lodging reduced the radiation-use efficiency of the crop by compressing the canopy. Similarly, Ward *et al*.^27^ reported that more light penetrates through a standing crop canopy than a lodged one. Any stress leading to a change in the supply of photosynthates can cause pod abortion or reduce the number of seeds in each pod^26^. Our results revealed that silique photosynthesis decreased significantly and linearly as lodging angle increased (Fig. 2). Artificially induced lodging forces the photosynthetic tissues (leaves and green pods) into positions that reduce the efficiency with which the crop is able to use the available light. A lower penetration of light in the lodged crop canopy reduces the overall radiation use efficiency. In lodged rapeseed, the upper pods easily become light saturated due to the limited photosynthetic capacity, whereas the lower pods receive low light levels, which reduce silique photosynthesis^26^. A lower silique photosynthetic rate ultimately reduces the available amount of assimilates for seed filling, thus resulting in lower seed weights. In the present study, seed and silique weight decreased as lodging angle increased, reaching a maximum reduction at S_10_ (Table 1).

In rapeseed, the leaf mainly serves as the photosynthetic organ before the initiation of flowering^28^. After 10–20 d of flowering, vegetative growth slows down and reproductive growth becomes more prominent, and siliques attain their maximum surface area. Simultaneously, due to senescence of leaves and shading from flowers and siliques, the silique photosynthesis exceeds that of leaves photosynthesis^26^. Assimilates derived from silique photosynthesis confirmed the role of siliques as a photosynthetic organ at the pod filling stage^29^. Any stress leading to a change in the supply of photosynthates can abort pods or reduce the number of seeds in each pod. Moreover, the silique is the only organ directly involved in the transportation of nutrients to the developing seeds^30^. In the present study, reductions in seed weight and yield by lodging might be linked to reduced silique photosynthesis and less assimilation of nutrients in the seeds. The potential of the crop to produce and remobilize photosynthetic assimilates to developing seeds is a factor determining seed weight. Seed weight increased with increase in remobilization and source-sink ratio during seed filling, leading to an increased seed yield, suggesting that seed weight primarily depends on resource availability^31^. A large proportion of the variation in seed weight is associated with environmental conditions during the critical period of seed filling. In lodged crop, canopy is compressed, thereby reducing radiation use efficiency, which limits photosynthesis in pods^26^.

In the present study, all yield components increased with increasing N application rates, consistent with the results of previous studies^32,33^. However, the optimum N application rate in winter rapeseed varied between 180–240 kg ha^−1^, depending on site conditions and the previous crop grown in the field^33,34^. Rapeseed has a high N demand, because its yield depends on several developmental stages^35,36^. A relatively high N application rate increases yield in rapeseed because total seed production is dependent upon the overall development of the rapeseed plant, leading to increased silique production. Nitrogen enhances the leaf area during flowering, increases the supply of assimilates to developing flowers and young siliques^37^, and leads to a higher number of siliques with a higher seed weight^38^.

The present results indicate that in all lodged and unlodged plants maximum seed yield was achieved at the combination of highest used density (45 plants m^−2^) and a N application rate of 270 kg ha^−1^, and the yield was not enhanced by increasing the N application rate further to 360 kg ha^−1^ (Tables 1 and 3). However, the increase in plant height and biomass was continuous with increasing N application rate. This indicates that at high N application rate, biomass investment in the stem might compete for resources with reproductive growth and yield determination traits. However, this maybe an acceptable compromise if it makes the stem stronger and improves lodging resistance. High N application rates decrease lignin deposition, vascular bundle area, and structural carbohydrate contents, resulting in poor stem and root stiffness in rice^39^. Root dry biomass and root diameter was reduced at high density and N rates. Root dry weight may play an important role for anchorage strength improvements. Root traits are more heritable; however, proper management practices for instance, reducing the actual plant population increases root lodging resistance and seed yield^40^. Root plate spread dimensions could be enhanced by reducing seed rate while maintaining high grain yields^41^. Lodging resistant varieties displayed greater stem diameter and dry biomass than the varieties susceptible to lodging^42^.

Khan *et al*.^12^ reported an increase in seed yield of rapeseed at higher planting density despite the lesser number of branches and pods per plant, and correlated it with increased lodging resistance due to reduced plant height and greater number of plants with uniform canopy at higher densities.

Lodging is a major constraint for yield performance under high-yielding conditions. In the present study, lodging caused a higher reduction in yield when induced at higher planting density (D_45_) and N application rate (N_360_) than at D_15_ and N_180_ (Table 3). Yield losses at higher N application rates could be attributed to excessive vegetative growth, as indicated in the present study by the highest plant height and aboveground biomass attained at the highest N application rate (Tables 4 and 5). Moreover, excessive N application delayed maturity, which is another reason for seed yield loss by lodging. In the present study, silique photosynthesis was reduced with increasing planting density (Fig. 2).

Seed yield loss at the highest plant density might be due to the reduced intensity of transmitted light and decreased silique photosynthesis. Furthermore, at higher planting density, the root neck diameter and root biomass were reduced, thereby decreasing the anchorage strength of the plants. Lower planting densities lead to thicker stem and greater resistance to lodging than at higher planting densities^41^. Moreover, our previous research indicated that increasing density from 30 to 60 plants m^−2^ decreased the lodging resistance because of the longer basal internode, higher centre of gravity, and decreased shoot diameter of plants^12^.

Correlations between lodging and agronomic traits are essential for developing strategies that might help breeders to improve resistance to both stem and root lodging, in addition to increasing grain yield. A major contributing factor towards lodging is the tallness of the plant. Traits with positive effects on stem and root strength, such as stem diameter, stem dry weight per unit length, and root dry weight showed positive associations with grain yield. Lodging resistance is correlated with the seed yield potential or yield performance, which are both characteristics of plant population rather than single plants and significantly interact with environmental factors. Better lodging resistance allows plants to benefit from high levels of soil fertility and favourable environments. Consequently, the yield can approach their yield potential^41^.

Seed filling is a crucial stage in oilseed growth, and lodging at this stage affects the oil quality, stability, and content. In addition, lodging leads to pod shattering and seed damage under field conditions, resulting in low oil quality^43^. Photosynthetic assimilates, such as sucrose, are converted into fatty acids during lipid biosynthesis^31^. Therefore, changes in the photosynthetic function or conversion of carbohydrates into lipids negatively affect the biosynthesis and accumulation of fatty acids. Our results indicated that lodging significantly reduced the oil content, which might be linked with reduced silique photosynthesis (Fig. 2, Table 6). The lodging treatments, which caused the greatest reduction in yield tended to have a greater impact on reducing the oil content^24^. Oil quality is associated with fatty acid composition, mainly the quantity of oleic, linoleic, and linolenic acids. The fatty acid composition of rapeseed oil usually consists of 60–65% oleic acid, 18–20% linoleic acid, 10–10.5% linolenic acid, and less than 3% erucic acid^37,44^. Glucosinolates are responsible for the bitter taste and pungent smell of the family Brassicaceae and its presence in rapeseed has been a major limitation for its use as animal feed^45^. According to Hu *et al*.^1^ the average glucosinolate level should be less than 35 μmol g^−1^ and that of erucic acid should be less than 3%. In the present study, glucosinolates and erucic acid contents were increased by lodging at 90° with maximum values reaching 33.2 μmol g^−1^ and 1.5% respectively. However, the values were within the safe limit. Lodging increased the content of stearic, palmitic, arachidic, and erucic acids, whereas it decreased the contents of linoleic and linolenic acids. Baylis and Wright^5^ reported that artificial lodging at 20° induced at the early pod filling stage of winter rapeseed reduced the oil content from 43 to 41.1% and increased the glucosinolate content from 12.8 to 13.4 μmolg^−1^ as compared with the supported control.

Our data revealed that lodging altered the fatty acid composition in rapeseed. The content of unsaturated fatty acids (oleic, linoleic, and linolenic acid) decreased, whereas that of saturated fatty acids (palmitic, stearic, and arachidic acid) increased with the increasing lodging angle (Table 6). Previous studies reported that fatty acid composition was affected by stress conditions; however, the effects of lodging on fatty acid composition have not been well documented. High temperature and drought stress during the seed filling stage in soybean and salinity stress in rapeseed altered the fatty acid composition by increasing oleic and palmitic acid contents and decreasing linoleic, linolenic, and stearic acid contents^20,21^. The oleic acid content can be strongly influenced by environmental conditions^44^. An increase in oleic acid content and decrease in linoleic and linolenic acid contents have been linked with the differential expression of fatty acid desaturases that control fatty acid conversion. In plants, fatty acid desaturase-2 is the most important enzyme for the synthesis of two polyunsaturated fatty acids: linoleic and linolenic acid^46^.

The synthesis of fatty acids is a light-dependent process. It utilizes ATP and reduces ions produced by photosynthesis. Seed filling is a sensitive stage of rapeseed, during which a change in temperature and solar radiation affects the fatty acid composition indirectly through the accumulation of photoassimilates and the modification of the source-sink ratio^47^. Ruuska *et al*.^23^ reported that seeds produced by plants grown under shaded conditions had a lower fatty acid content as compared with that in seeds of plants fully exposed to sunlight, and thus, shading due to lodging might affect fatty acid composition. In the present study, the oil, glucosinolate, and linoleic acid contents decreased whereas protein and stearic acid contents increased at higher N rates. The negative relationship between oil and protein content has been previously reported in winter rapeseed, because the protein content increases at the expense of oil content^33,37,48^. Behrens^49^ indicated that oleic acid content increased, while that of linoleic, linoleic, and erucic acids decreased as the N application rate increased.

These results indicated that major contributing factors for yield loss under higher N fertilizer treatment are the taller plants with more aboveground biomass and lesser increase in root diameter and root biomass. Therefore, lodging-induced seed yield losses might be reduced by selecting the optimum density and N application rates at which plants have optimum height, shorter basal internodes, and uniform canopy with high light interception traits that are associated with higher lodging resistance. Additional research on balanced N fertilizer management should be conducted to determine more powerful strategies for minimizing lodging risks while maintaining relatively high seed yields

## Conclusions

Our study demonstrated that lodging at the seed filling stage of rapeseed reduced yield, and the maximum reduction was observed at a lodging angle of 90°. A significant yield reduction (6–13%) was obtained when 30° lodging was induced at an early pod-filling stage (10 d post-flowering), with relatively less reduction at the 20 (3–8%) and 30 d post-flowering (0–2%) stages. Maximum seed yield was recorded at N_270_D_45_. In lodged plants, seed yield loss was higher at the highest N rate, with a maximum yield loss (28–32%) found at N_360_D_45_. Lodging altered the fatty acid composition by decreasing linoleic and linolenic acid contents and increasing stearic, palmitic, and arachidic acid contents. The oil quantity and quality were also affected as indicated by the lower oil content and the higher protein, erucic acid, and glucosinolate contents. A decrease in silique photosynthesis indicated that the alteration of fatty acids might be due to the limited availability of sugars in the plant and translocation of sugars from the leaves to the seeds during the seed filling stage. However, further research is needed to understand the underlying physiological mechanisms that control lodging in rapeseed better.

## Materials and Methods

### Site characteristics and field trial management

Field experiments were conducted in Wuhan, Hubei Province (114°22′E, 30°29′N) during the 2014/2015, 2015/2016, and 2016/2017 growing seasons. The local subtropical monsoon climate is characterized by cold winters (November–February) and hot summers (July–September). Information on the average monthly temperature and rainfall in the 2014–2017 growing seasons is presented in Figure S1. The soil organic matter, available and total N, phosphorus and potassium, and pH of the topsoil (20 cm) were determined as described by Bao^50^ are presented in Table S1. Total N was determined by the Kjeldahl method^51,52^. The available N content was determined using 1 M potassium chloride (KCl) extraction, followed by colorimetric analysis^45^. Available P was determined by the Olsen method according to Black^53^, organic content by the titrimetric method^54^, and available K with a flame photometer^55^. The high-yield winter rapeseed cultivar Huayouza 62 was manually sown in rows on October 4 in 2014, 2015, and 2016. The previous crop sown in the plots each growing season was rice. During experiment to control weeds, 50% acetochlor was sprayed after sowing; 10% quizalofop-p-ethyl was sprayed at 2–4 leaves stage; and 50% benazolin was sprayed at 5–6 leaves stage. To control aphid, 50% Imidacloprid was sprayed when aphid strain rate was 8%. Fungicide 40% dimethachlon 0.1–0.2 kg/40–50 L water was sprayed at the beginning of flowering to control *Sclerotinia sclerotiorum*.

### Identification of the critical pod-filling stage

In the 2014/2015 growing season, plants were sown at three different plant densities (D_15_, 15 plants m^−2^; D_30_, 30 plants m^−2^; and D_45_, 45 plants m^−2^) and fertilized with three different N rates (N_180_, 180 kg N ha^−1^; N_270_, 270 kg N ha^−1^; and N_360_, 360 kg N ha^−1^). The ranges of density (15–45 plants m^−2^) and N rates (180–360 kg ha^−1^) were selected based on commonly used practices in the Yangtze River Basin. Nitrogen was broadcast as urea fertilizer (46% N) in three split doses: 50% before sowing, 30% during the over-wintering period, and 20% during the bud development period. Each subplot dimension was 8 m × 2 m, with 30 cm between the rows. A single lodging angle (30°) was manually induced by pushing the plants with canes at pod filling stage (BBCH 69) of rapeseed. Lodging angle was induced three times with interval of 10 days, at 10 d post flowering (S_10_), 20 d post flowering (S_20_) and 30 d post flowering (S_30_). The plants remained lodged until harvest.

### Identification of the critical lodging angle

In the 2015/2016 and 2016/2017 growing seasons, plants were sown at three different plant densities (D_15_, 15 plants m^−2^; D_30_, 30 plants m^−2^; and D_45_, 45 plants m^−2^) and fertilized with three different N rates (N_180_, 180 kg N ha^−1^; N_270_, 270 kg N ha^−1^; and N_360_, 360 kg N ha^−1^). Four lodging angles (0°, 30°, 60°, and 90°) were manually induced at the critical pod-filling stage (S_10_, 10 d post flowering); supported control plants were at 0° and completely horizontal plants were at 90° (Figure S2). The plants remained lodged until harvest.

### Data collection

#### Humidity and air temperature

Humidity and air temperature at the ground (10 cm above soil surface) and canopy level of crop was monitored using Thermo-Hygrometer (8718) at pod filling stage from plots lodged at 0°, 30°, 60°, and 90° in April 2016.

#### Morphological traits, yield and yield related attributes

On May 20, 2015, May 15, 2016, and May 15, 2017, five plants at maturity were randomly selected and uprooted from each plot to determine plant height, root neck diameter, number of pods per plant, number of seeds per plant, and the weight of 1,000 seeds. Plant height was measured from the stem base to the highest bud. The roots were cut from the shoot, and the root neck diameter was measured at the cotyledonary scar using Vernier calipers. The shoots and roots were oven-dried at 72 °C to a constant weight to estimate the biomass. The remaining plants in each plot were harvested to measure seed yield. Seeds were dried, weighed, and seed yield was estimated in tonnes per hectare (t ha^−1^).

#### Yield losses

Lodging-induced yield loss was calculated by subtracting the observed yield from the yield in the absence of lodging. Yield losses were expressed in percentages using a formula:

1 $$Y\,loss=Y\,att-Y\,act/Y\,att\times 100$$

where Y loss is the percent yield loss of rapeseed per hectare (t ha^−1^), Yatt is attainable yield, and Yact is actual yield.

#### Silique photosynthesis

Silique photosynthesis was recorded at 20^th^ d after flowering ending in 2015/16. Siliques (pod containing seeds) were selected from the middle of the main inflorescence from plants which were induced with different lodging angles (0°, 30°, 60° and 90°). The silique photosynthesis index (*P*_*n*_, µmol m^−2^ s^−1^) was estimated using LI-6400 portable photosynthesis system (LI- 6400-07, Licor, Lincoln, NE, USA) between 9:30 am and 1:30 pm.

#### Oil contents and seed quality

Seed quality parameters were measured at S_30_. The oil content was analyzed by nuclear magnetic resonance (NMR, mq-20, Bruker, Germany)^56^, protein content using Kjeldahl method and glucosinolates by using ultra performance liquid chromatography (UPLC) method as described by Gratacós-Cubarsí *et al*.^57^, and fatty acid composition by near-infrared reflectance spectroscopy (NIRS; Foss NIR Systems Inc., USA) using standard methods^58^.

### Statistical analyses

Field experiments were carried out as randomized complete block designs with four replications per block. Analysis of variance (ANOVA) in conjunction with Duncan’s multiple range test was applied to identify significant differences between treatment levels and combinations of treatments at p < 0.05. All analyses were carried out using SAS 8.1 (SAS Corp., Cary, NC, USA), and graphs were constructed using Microsoft Excel 2010 (Microsoft Corp., Redmond, WA, USA). The difference in data for two years and interaction between the year, density and nitrogen were not significant (p > 0.05) for oil content and fatty acid composition, therefore, only main effects were shown and data were averaged for two years.

## Electronic supplementary material

Supplementary Information
