# Supplementary material for: Alteration in yield and oil quality traits of winter rapeseed by lodging at different planting density and nitrogen rates
Source: Sci Rep. 2018 Jan 12;8:634. doi: 10.1038/s41598-017-18734-8 (PMC5766575; doi:10.1038/s41598-017-18734-8)
Supplement: Supplementary file 1 — Supplementary Information [file 41598_2017_18734_MOESM1_ESM.pdf]

Alteration in yield and oil quality traits of winter rapeseed by lodging at different planting density and nitrogen rates

Shahbaz Khan[1,a], Sumera Anwar[1,a], Jie Kuai[1], Ali Noman[2], Muhammad Shahid[1], Mairaj Din[3], Ahmed Ali[1], Guangsheng Zhou[1,]*

Affiliation [1]College of Plant Science and Technology, Huazhong Agricultural University, Wuhan, Hubei Province, P.R. China

[2]Government College University, Faisalabad, Pakistan

[3]College of Resources and Environment, Huazhong Agricultural University, Wuhan, Hubei Province, P.R. China

[a]Equal contribution

*Correspondence: zhougs@mail.hzau.edu.cn

Table S1

Soil fertility in field experiments conducted in Wuhan, Hubei Province, China, during the growing seasons of 2014-2017.

Soil samples collected	Texture	pH	Organic matter ($g\ kg^{-1}$)	Available N ($mg\ kg^{-1}$)	Available P ($mg\ kg^{-1}$)	Available K ($mg\ kg^{-1}$)	Total N (%)	Total P (%)	Total K (%)
2014/15	silt clay loam	6.20	8.51	62.5	9.80	145	0.06	0.03	1.10
2015/16	silt clay loam	6.15	8.01	64.5	9.81	148	0.07	0.03	1.11
2016/17	silt clay loam	6.22	8.40	72.4	10.30	145	0.09	0.04	1.40

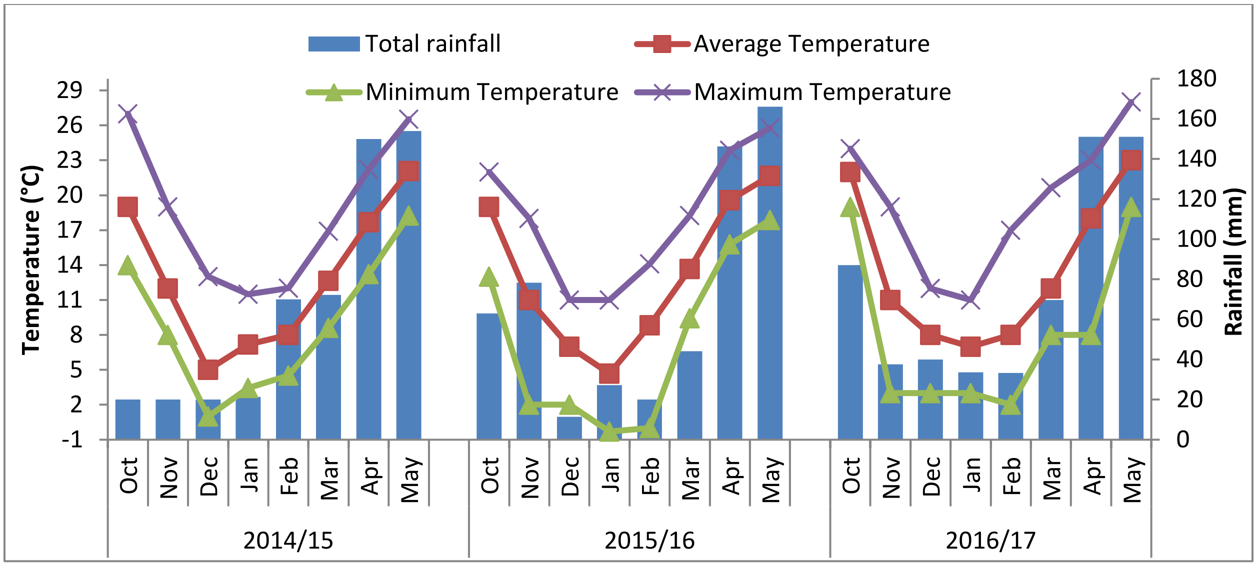

Figure S1. Monthly total rainfall and monthly temperature during the crop growing season in the experiment conducted during 2014-2017.

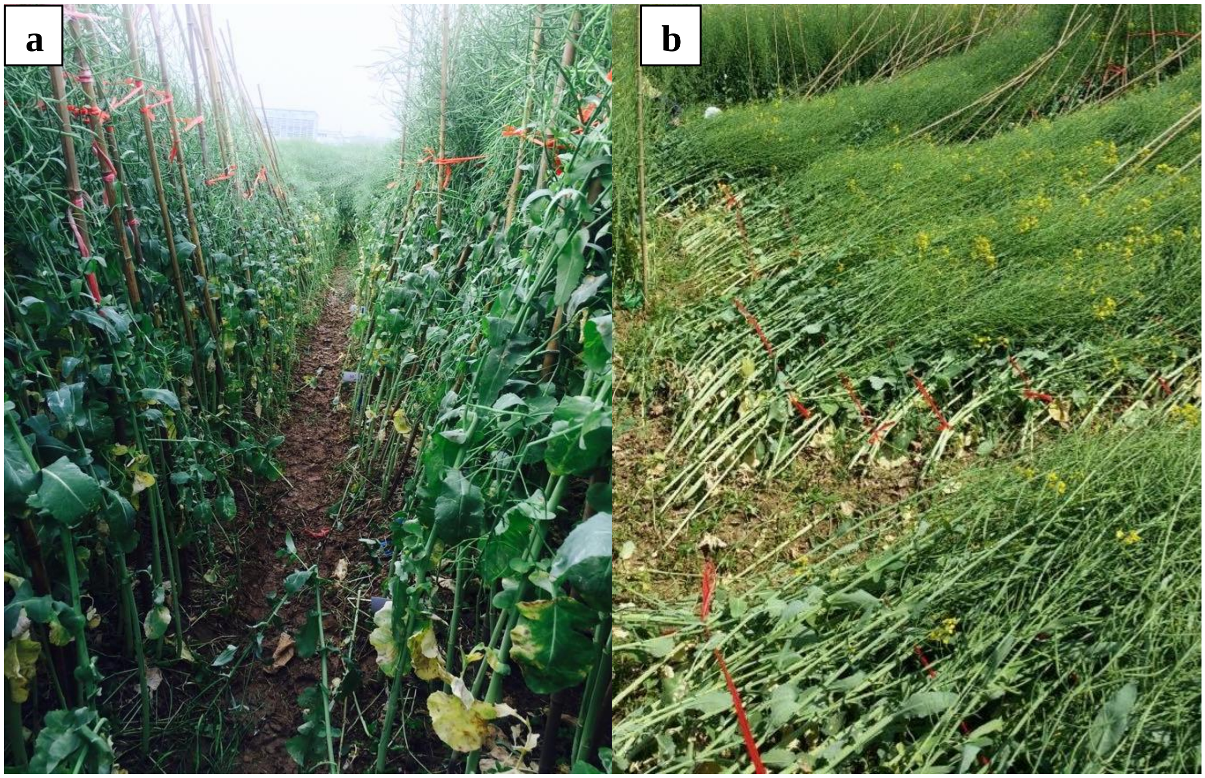

Figure S2. The induction of different artificial lodging angles during field trial of rapeseed at Huazhong Agricultural University, a) supported control, b) lodged plants
